# A Molecular Probe with Both Chromogenic and Fluorescent Units for Detecting Serine Proteases

**DOI:** 10.3390/molecules26020482

**Published:** 2021-01-18

**Authors:** Kirara Ishida, Yushi Nakamura, Tetsuo Ohta, Yohei Oe

**Affiliations:** Department of Biomedical Sciences and Informatics, Faculty of Life and Medical Sciences, Doshisha University, Kyotanabe, Kyoto 610-0394, Japan; ctud1009@mail4.doshisha.ac.jp (K.I.); yusnakam@mail.doshisha.ac.jp (Y.N.); tota@mail.doshisha.ac.jp (T.O.)

**Keywords:** molecular fluorescence probe, chromogenic molecular probe, protease, analysis

## Abstract

A molecular probe with l-phenylalanine *p*-nitroanilide and l-lysin 4-methylcoumaryl-7-amide, in which these amino acid derivatives are connected through a succinic-acid spacer, was prepared. Trypsin and papain were detected by blue-fluorescence emission of generated 7-amino-4-methylcoumarin (AMC). α-Chymotrypsin and nattokinase were detected from both the blue-fluorescence emission of AMC and the UV absorbance of *p*-nitroaniline. In addition, different time courses of *p*-nitroaniline and AMC were observed between the reaction of **P1** with α-chymotrypsin and that with nattokinase. In the case of nattokinase, both the fluorescence emission and UV absorbance slowly increased. In contrast, the increasing UV absorbance was saturated at the early stage of the reaction of the present probe with chymotrypsin, whereas the fluorescence emission continuously increased in the following stages.

## 1. Introduction

Proteases are enzymes that catalyze the hydrolysis of peptide bonds in proteins and are implemented in the development of many pathological conditions [1,2,3]. Methods for protease assays include liquid chromatography, electrochemistry, mass spectrometry, and fluorescence [4,5,6,7,8,9]. A fluorescence assay is one of the most popular among them due to its rapid responsibility and high sensitivity [4]. As for a fluorescence assay, “turn-on” molecular probes are generally utilized. The fluorescence of fluorophores in these molecular probes is initially quenched (“off” state), and the fluorescence turns on when the probes are reacted with serine proteases (“on” state). FRET [4] and the amide-bond formations between the nitrogen atom of fluorophores such as 7-amino-4-methylcoumarin (AMC) and the carboxy group of amino acid residue [5] have been utilized to achieve the “off” state. Being similar to fluorescent molecular probes, chromogenic molecular probes, which sometimes use *p*-nitroaniline as a chromogenic substrate, have also been reported [10,11,12,13,14,15,16,17]. In general, to detect the enzyme selectively, amino acid residues or peptides have been used. For example, phenylalanine was used as an amino acid moiety in the “turn-on” probe to detect α-chymotrypsin, which is known to cleave the amide (or peptide) bond at the C-terminus of tyrosine, tryptophan, and phenylalanine selectively [7,10]. Moreover, lysin was utilized in a peptide FRET probe to detect trypsin activity [15,16]. These designed molecular probes could measure the target enzymatic activities; however, untargeted enzymes sometimes reacted with the molecular probe due to their poor substrate selectivity [18]. In this work, we focused on such unexpected enzymatic activity in the reaction of proteases with the “turn on” fluorescence and/or chromogenic molecular probes.

**Figure 1:**
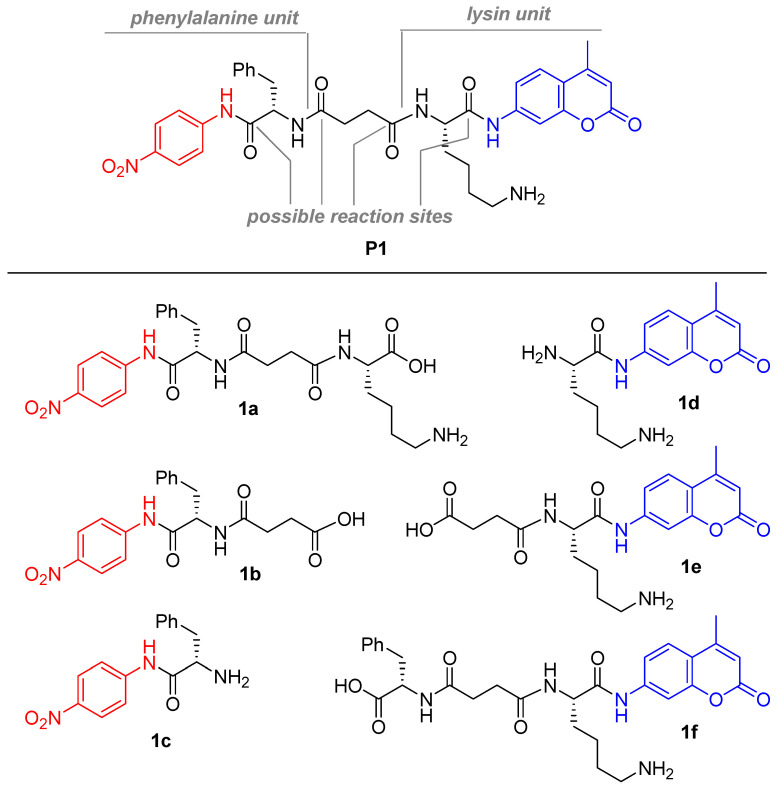
Designed molecular probe (**P1**) and plausible fragment probes **1a**–**f**.

Our designed molecular probe, **P1** hereafter, is shown schematically in Figure 1. Both a chromogenic-probe and a fluorescence-probe are incorporated in **P1**. In **P1**, *p*-nitroaniline is released when the corresponding amide bond in the phenylalanine unit is cleaved by an enzyme that reacts with hydrophobic substrates like chymotrypsin. Moreover, AMC is released from the lysin unit by the reaction with an enzyme such as trypsin that reacts with hydrophilic substrates. These two units are connected through the succinic acid moiety. Installing the two molecular-probe units in one molecule brings the following advantage. **P1** has four possible reaction sites with proteases, which can generate six different fragment probes with different sizes and hydrophilicities. It is therefore considered that various timings to release the pigment and fluorescent moieties can be provided according to the substrate selectivity of the enzymes. We hypothesized that the use of **P1** would distinguish the activity of proteases in more detail than a similar molecular probe with only one unit of the two in the proposed probe. Hereafter, the synthesis of the new molecular probe with the two different molecular-probe units is described, and its reactivity with several serine proteases is demonstrated.

## 2. Results and Discussion

### 2.1. Synthesis of ***P1***

**Scheme 1:**
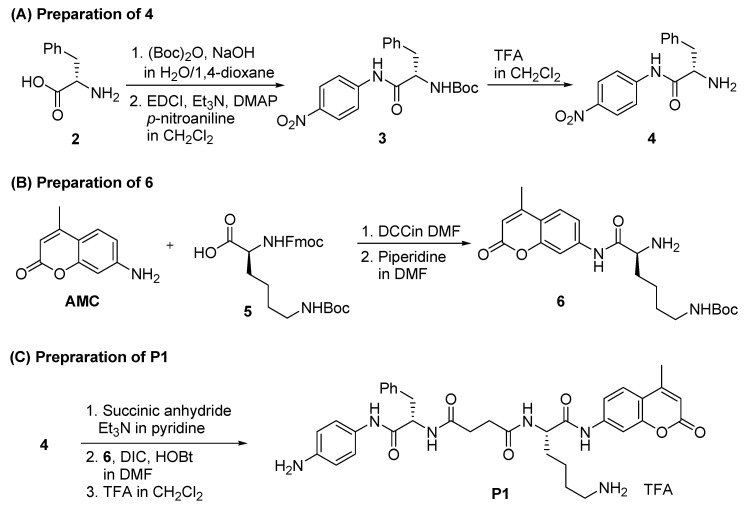
Synthesis of **P1.**

As shown in Scheme 1, **P1** can be synthesized by preparing *p*-nitrophenylanilide **4** (phenylalanine unit) and AMC anilide **6** (lysin unit) and connecting both units to a succinic acid spacer. Initially, anilide **4** was prepared from commercially available l-phenylalanine (**2**) through the three steps (Scheme 1A). l-Phenylalanine is thus *N*-Boc protected with (Boc)_2_O under basic conditions. The obtained *N*-Boc-phenylalanine is then condensed along with *p*-nitroaniline in the presence of EDCI, Et_3_N, and DMAP to give *N*-Boc-l-phenylalanine *p*-nitroanilide **3** (30% yield in two steps). The obtained *N*-Boc-l-phenylalanine *p*-nitroanilide (**3**) was then treated with trifluoroacetic acid (TFA) in CH_2_Cl_2_ to remove the Boc protecting group and afford the desired anilide (**4**) at 87% yield.

Next, the lysin unit was prepared (Scheme 1B). Thus, ε-Boc-α-Fmoc-l-lysin (**5**) was reacted with AMC [19] in the presence of DCC in DMF to give the corresponding adduct in 44% yield, which was then treated with piperidine in DMF to afford lysin unit **6** at a yield of 75%. To synthesize the probe **P1**, both prepared units **4** and **6** were connected through a succinic acid spacer (Scheme 1C). Thus, the anilide **4** was reacted with succinic anhydride in the presence of Et_3_N in pyridine to afford the corresponding adduct, which was then reacted with unit **6** in the presence of DIC and HOBt in DMF to give *N*-Boc-protected probe. Finally, the product was treated with TFA in CH_2_Cl_2_ at ambient temperature to provide **P1** as a TFA adduct.

### 2.2. UV and Fluorescence Spectra of ***P1***

**Figure 2:**
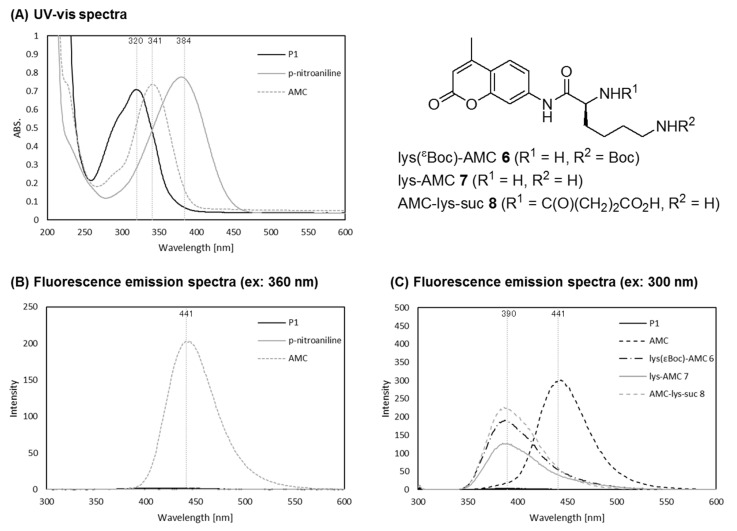
UV-vis and fluorescence emission spectra of *p*-nitroaniline, AMC, **P1** and AMC anilides.

Once the desired probe **P1** was obtained, UV-vis absorbance and fluorescence properties of *p*-nitroaniline, AMC, and **P1** were investigated (Figure 2). As shown in Figure 2A, both absorptions derived from *p*-nitroaniline (at 384 nm) and AMC (at 341 nm) are shifted, and λ_max_ of **P1** appeared at 320 nm. It is considered that these blue shifts are caused by the formation of amide bonds by chromophores in *p*-nitroaniline and AMC with phenylalanine and lysin, respectively. The solution of **P1** for these spectra was colorless; therefore, it is expected that the release of *p*-nitroaniline by the reaction of **P1** with proteases could also be visually confirmed by the color change of the solution. As shown in Figure 2B, the fluorescence-emission spectra of *p*-nitroaniline, AMC, and **P1** excited at 360 nm reveal that the fluorescence of AMC moiety in **P1** is highly suppressed. To clarify the reason for this effective suppression of fluorescence emission, several AMC derivatives were prepared, and their fluorescence properties were investigated. Fluorescence emission spectra excited at 300 nm are shown in Figure 2C. The maximum emission of AMC itself appears at a wavelength of 441 nm. In contrast, AMCs connected with lysin (lys-AMC **7**) or lysin derivatives (**6** and **8**) show maximum fluorescence emission at wavelengths of 387, 390, and 390 nm, respectively. These blue shifts of the emission peaks are caused by the formation of amide bonds between the amino group of AMC and the carboxylic group of lysin. It is noteworthy that almost no fluorescence emission was observed with **P1** excited at 300 nm. It is considered that this quenching of fluorescence emission was caused by absorption of fluorescence energy by *p*-nitroanilide. Fluorescence emission of **P1** was therefore effectively suppressed by both the amide-bond formation and the FRET effect.

### 2.3. Reaction of ***P1*** with Proteases

**Figure 3:**
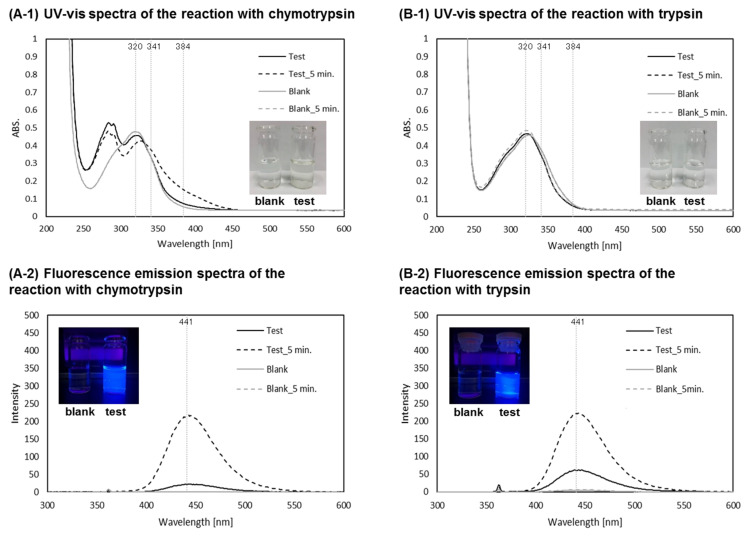
UV-vis and fluorescence emission spectra (excited at 360 nm) of the test and blank samples with α-chymotrypsin (**A**) or trypsin (**B**).

The reaction of **P1** with chymotrypsin and trypsin was preliminarily investigated. **P1** was reacted with α-chymotrypsin (4.8 U/mL) in pH 8.0 Tris-HCl buffer solution. A test solution (with α-chymotrypsin) and a blank solution (without α-chymotrypsin) were prepared, and the UV-vis and fluorescence emission spectra of both solutions were recorded immediately after the preparation and after 5 min. According to the spectra, absorbance at 320 nm derived from **P1** slightly decreased after 5 min, while absorbance at 384 nm derived from *p*-nitroaniline was increased (Figure 3(A-1)). The reaction solution turned slightly yellow, indicating that *p*-nitroaniline was released into the reaction solution. On the contrary, the UV-vis spectra and color of the blank solution did not change. As for both the fluorescence-emission spectra of the test solution immediately after the preparation and after 5 min, the fluorescent intensities at 441 nm apparently increased, suggesting that AMC was apparently released under the described reaction conditions despite the fact that α-chymotrypsin is known to cleave the amide bond at the *C*-terminus of hydrophobic amino acid residue selectively (Figure 3(A-2)). It is considered that the amide bond at the *C*-terminus of lysin in **P1** was recognized as a reaction site by α-chymotrypsin due to the hydrophobicity of the AMC and/or the probe molecule itself. The release of AMC was confirmed visibly under UV irradiation (365 nm).

The reaction of **P1** with trypsin (4.8 U/mL) gave a different result from that with α-chymotrypsin. **P1** was treated with trypsin in PBS buffer at pH 7.4. In a similar manner to the reaction of **P1** with α-chymotrypsin, a test solution (with trypsin) and a blank solution (without trypsin) were prepared, and the UV-vis and fluorescence emission spectra of both solutions were recorded immediately after the preparation and after 5 min. The UV-vis spectrum suggested that *p*-nitroaniline was not obtained either immediately after the preparation of the test solution or 5 min after the preparation (Figure 3(B-1)). The solution was colorless even after 5 min, indicating that *p*-nitroaniline was not released into the reaction solution. Moreover, absorbance at 341 nm hardly increased, indicating the generation of AMC. The fluorescence-emission spectrum (excited at 360 nm) clarified the generation of AMC by the apparent increase of fluorescent intensity at 441 nm (Figure 3(B-2)). Although several molecules, including the lysin unit, are known as substrates for the trypsin, α-chymotrypsin also reacted with the lys-AMC unit of **P1** in this case. In other words, chymotrypsin is hardly distinguishable from trypsin by the present fluorescence results. The phenylalanine unit supports to solve this issue. *p*-Nitroaniline was released when **P1** was reacted with α-chymotrypsin, whereas no *p*-nitroaniline was obtained with trypsin. Therefore, **P1** can distinguish between α-chymotrypsin and trypsin by the combination of UV adsorption with fluorescence.

**Figure 4:**
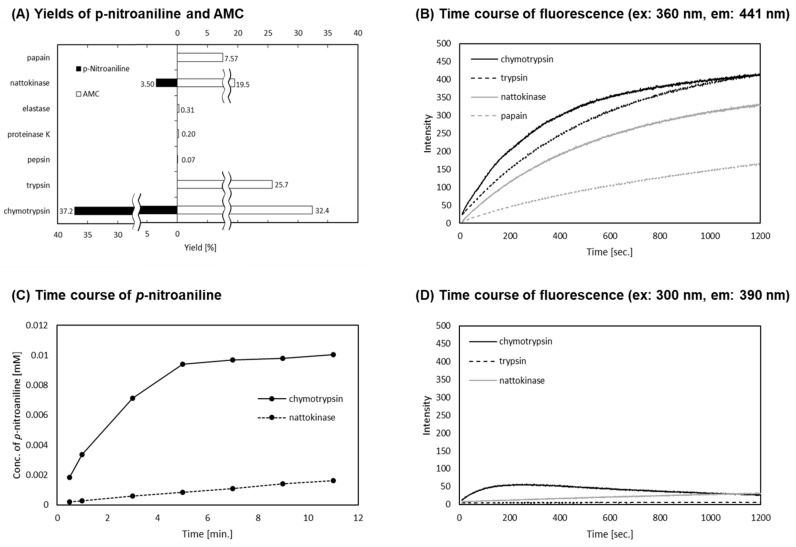
Quantitative analysis of the reaction of **P1** with proteases.

**P1** was then reacted with α-chymotrypsin, trypsin, pepsin, proteinase K, elastase, nattokinase, and papain (4.8 U/mL) for 5 min, and the amounts of obtained *p*-nitroaniline and AMC were calculated by using high-performance liquid chromatography (HPLC) and fluorescence-emission spectra, respectively. Yields of the obtained *p*-nitroaniline and AMC are shown in Figure 4A. Both α-chymotrypsin and nattokinase cleaved the amide bond at the *C*-terminus of l-phenylalanine in **P1** to give *p*-nitroaniline at yields of 37.2 and 3.5%, respectively. This result is not surprising since it is known that nattokinase is a subtilisin endopeptidase and selectively cleaves the *C*-terminus of hydrophobic amino acid residue in general. Trypsin, elastase, papain, and pepsin, which is known to cleave the peptide at the *C*-terminus of small aliphatic, basic, and acidic amino acid residues, respectively, and, as expected, no *p*-nitroaniline was detected after 5 min. Furthermore, no *p*-nitroaniline was detected in spite of the wide substrate scope of proteinase K (presumably due to the unsuitable reaction conditions for this enzyme). On the contrary, both the expected and unexpected results were observed by the fluorescence emission spectra. Thus, AMC was released when **P1** was reacted with α-chymotrypsin, trypsin, nattokinase, and papain. Trypsin and papain are known to cleave the peptide bond of basic amino acid residue; therefore, AMC was released as expected. α-Chymotrypsin and nattokinase, which usually react with hydrophobic amino acid residue, also afforded AMC presumably due to the hydrophobic property of **P1**. Only a little amount of AMC was obtained when elastase and pepsin were used. These results can be understood in terms of the substrate selectivity of these two enzymes. Fluorescent intensity was almost zero when proteinase K was used. These overall observations suggest that the balance between the obtained *p*-nitroaniline and AMC after 5 min can partially explain the selectivity of proteases. Thus, **P1** can classify the seven proteases tested in this work into three categories. α-Chymotrypsin and nattokinase were classified into category I, which react with both phenylalanine and lysin units. Trypsin and papain were classified into category II since these two proteases were reacted only with the lysin unit. Proteases in category III, elastase, proteinase K and pepsin, obtained only a little fluorescence emission. In addition, it is considered that category I could be subdivided by the ratio of *p*-nitroaniline to AMC. The ratio is 1.15 in the case of α-chymotrypsin, whereas the ratio is only 0.18 in the case of nattokinase. This result suggested that nattokinase reacted with the lysin unit more selectively compared with α-chymotrypsin. Therefore, we considered that **P1** is more useful for the categorization of proteases than the molecular probe with a single reporter unit, which classifies these enzymes into two groups (reactive or unreactive). The time courses of the yield of *p*-nitroaniline and fluorescence intensities are shown in Figure 4B–D. Fluorescence emissions (excited at 360 nm) are continuously increased with proteases in category I and II in 20 min (Figure 4B). Category I proteases, α-chymotrypsin and nattokinase, obtained *p*-nitoroaniline (Figure 4C). With α-chymotrypsin, *p*-nitroaniline was obtained rapidly and almost saturated after 5 min. The time course of fluorescence-emission spectra (excited at 300 nm; emission at 390 nm) with α-chymotrypsin is shown in Figure 4D, where the fluorescent intensity was smoothly increased until 5 min later. This time course is very similar to that of *p*-nitroaniline, indicating the generation of fragment **1f** (shown in Figure 1) by selective cleavage of the *p*-nitroanilide moiety at the initial stage of the reaction of **P1** with α-chymotrypsin. Interestingly, the fluorescent intensity excited at 300 nm was decreased after this (Figure 4D), suggesting that the fragment molecular probe **1f** generated in situ was reacted with α-chymotrypsin. We considered that a more detailed classification of proteases would be possible by combining the categorization described above and the investigation of time courses of the enzymatic reactions by use of **P1**.

**Figure 5:**
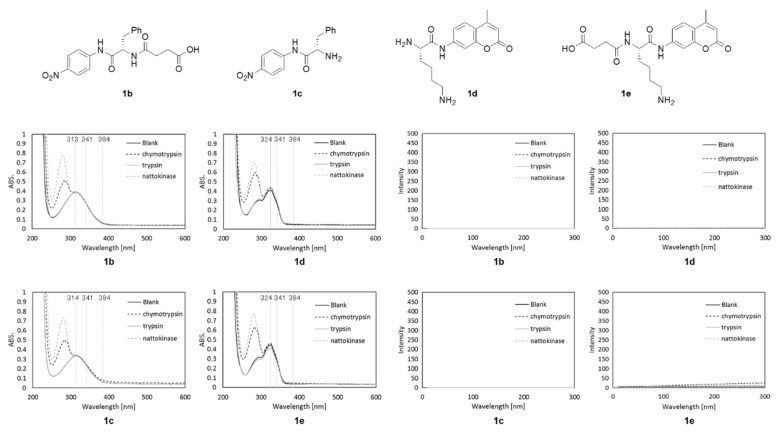
UV-vis and fluorescence emission spectra (excited at 360 nm) of the reaction mixture of fragment probe **1b**–**e** with proteases. Enzymes showed their absorbance maxima at 280–290 nm (see Supporting Information).

Finally, several fragment molecular probes (**1b**–**e**) were reacted with α-chymotrypsin, trypsin, and nattokinase. UV-vis and fluorescence-emission spectra of these reaction mixtures are shown in Figure 5. Although very weak fluorescence emissions were observed when **1e** was used as a substrate for the enzymatic reactions, almost no enzymatic reactions took place with fragment molecular probes **1b**–**d**. These results suggested that **P1** was a more suitable substrate than these fragment probes for the proteases tested here and that combination of the tests by using **1b**–**d** independently cannot classify the proteases in the same way as using **P1**.

## 3. Materials and Methods

### 3.1. General Methods

^1^H and ^13^CNMR spectra were recorded with a Bruker Ascend 400 spectrometer (Parque Tecnológico de AndalucíaC/Severo Ochoa, Málaga, Spain, 400 MHz for ^1^H and 100 MHz for ^13^C) using TMS (δ = 0 ppm) as an internal standard for ^1^H NMR and CDCl_3_ (δ = 77 ppm) or DMSO-*d_6_* (δ = 39.7 ppm) as that for ^13^C NMR spectroscopy (Parque Tecnológico de AndalucíaC/Severo Ochoa, Málaga, Spain). High-resolution mass spectra (FAB) were recorded by using a JEOL JMS-700 instrument (Musashino Akishima, Tokyo, Japan)with *m*-nitrobenzyl alcohol as the matrix and PEG-200 as the calibration standard. UV-vis spectra were recorded on a Shimadzu UV-2600 spectrometer in the wavelength range of 200–600 nm (Tokyo, Japan). Fluorescence emission spectra were recorded on a Shimadzu RF-5300 in the wavelength range of 300–600 nm (Tokyo, Japan). Excitation and emission wavelengths were, respectively, 300 and 390 nm or 360 and 430 nm. All fluorescence emission spectra were recorded bandwidths of 1.5 nm for excitation and 1.5 nm for emission. HPLC analysis was performed on a Shimadzu LC system (Tokyo, Japan, pump: LC-20AD, column oven: CTO-20A, UV detector: SPD-20AV at 370 nm, and degasser: DGU-20A3) with a Shimadzu VP-ODS column. The mobile phase was MeOH/H_2_O (2/3 *v*/*v*), the flow rate was 0.75 mL/min, and the column temperature was kept at 40 °C.

### 3.2. Synthesis of Substrates

#### 3.2.1. Synthesis of *N*-(Boc)-l-phenylalanine p-nitroanilide (**3**)

To a 100 mL round-bottomed flask, dichloromethane (30 mL), *N*-(Boc)-l-phenylalanine (1.325 g, 5.0 mmol), EDCI (1.525 g, 8.0 mmol), and Et_3_N (1.280 g, 12.5 mmol) were added. Then, *p*-nitroaniline (0.693 g, 5.0 mmol) and DMAP (0.625 g, 5.0 mmol) were added to the mixture with stirring. The reaction mixture was stirred at ambient temperature overnight. The solvent was removed, and the residue was dissolved in ethyl acetate. The organic phase was washed with 10% NaHCO_3_ aq. and dried with Na_2_SO_4_. After filtration, the filtrate was evaporated to afford the crude product as a solid, which was then washed with diethyl ether to give anilide **3** (0.651 g, 34% yield).

*N*-(Boc)-l-phenylalanine *p*-nitroanilide (**3**): ^1^H NMR (400 MHz, CDCl_3_) δ = 8.50 (brs, 1H, -N*H*CO_2_), 8.18 (d, *J* = 9.2 Hz, 2H, Ar-*H*), 7.57 (d, *J* = 9.2 Hz, 2H, Ar-*H*), 7.24–7.35 (m, 5H, -C_6_*H*_5_), 5.04 (brs, 1H, -N*H*C(O)-), 4.46–4.50 (m, 1H, -NHC*H*(Bn)C(O)), 3.12–3.22 (m, 2H, Ph-C*H*_2_-), 1.44 (s, 9H, -C(C*H*_3_)_3_) ppm. [20]

#### 3.2.2. Synthesis of l-phenylalanine p-nitroanilide (**4**)

To a 100 mL round-bottomed flask anilide **3** (0.346 g, 0.9 mmol) and dichloromethane (30 mL) were added. TFA (4 mL) was added to the solution, and the reaction mixture was stirred at ambient temperature for 1 h. After the removal of volatiles, ethyl acetate was added to the residue. The organic layer was neutralized with 10% Na_2_CO_3_ aq., washed with brine and then dried with Na_2_SO_4_. After filtration, the filtrate was evaporated, and the residue was dried in vacuo to afford l-phenylalanine *p*-nitroanilide (**4**) (0.223 g, 87% yield).

l-phenylalanine *p*-nitroanilide (**4**): ^1^H NMR (400 MHz, CDCl_3_) δ = 9.91 (s, 1H, -CO_2_*H*), 8.23 (d, *J* = 9.2 Hz, 2H, Ar-*H*), 7.78 (d, *J* = 9.2 Hz, 2H, Ar-*H*), 7.24–7.37 (m, 5H, -C_6_*H*_5_), 3.79 (dd, *J* = 4.0 Hz, 9.2 Hz, 1H, Ph-C*H*_2_-), 3.38 (dd, *J* = 4.0 Hz, 14 Hz, 1H, Ph-C*H*_2_-), 2.83 (dd, *J* = 9.6 Hz, 13.6 Hz, 1H, -NHC*H*(Bn)C(O)) ppm. [21]

#### 3.2.3. Synthesis of α-Fmoc-ε-Boc-l-lysin 4-methylcoumaryl-7-amide

To a 100 mL round-bottomed flask, *N*-*α*-Fmoc-*N*-*ε*-*t*-Boc-l-lysin (3.097 g, 6.66 mmol), dichloromethane (40 mL), and DCC (1.3986 g, 6.66 mmol) at 0 °C were added, and the reaction mixture was stirred at 0 °C. After 1 h, AMC (1.1681 g, 6.66 mmol) was added to the solution, and the solution was stirred at ambient temperature overnight. The precipitates were filtered off, and the filtrate was dried under reduced pressure. The obtained solid was washed with hexane to afford *α*-Fmoc-*ε*-Boc-l-lysin 4-methylcoumaryl-7-amide (1.833 g, 44% yield).

*α*-Fmoc-*ε*-Boc-l-lysin 4-methylcoumaryl-7-amide: ^1^H NMR (400 MHz, DMSO) δ = 10.48 (s, 1 H, N*H*), 7.89 (d, *J* = 7.6 Hz, 2 H, Ar-*H*), 7.78–7.72 (m, 5 H, Ar-*H*, N*H*), 7.50 (d, *J* = 8.8 Hz, 1 H, Ar-*H*), 7.42 (t, *J* = 4.9 Hz, 2 H, Ar-*H*), 7.32 (t, *J* = 4.9 Hz, 2 H, Ar-*H*), 6.78 (s, 1 H, N*H*), 6.27 (s, 1 H, Ar-*H*), 4.29 (m, 2 H, -OC*H*_2_CH-), 4.26 (d, *J* = 12.8 Hz, 1 *H*, -OCH_2_C*H*-), 4.30 (s, 1 H, -NHC(O)C*H*-), 2.90 (s, 2 H, -C(O)NHC*H*_2_-), 2.40 (s, 3 H, -C*H*_3_), 1.74–1.15 (m, 13 H, -CHC_3_*H*_6_-, -C(C*H*_3_)_3_) ppm. [22]

#### 3.2.4. Synthesis of ε-Boc-l-lysin 4-methylcoumaryl-7-amide (**6**)

To a 100 mL round-bottomed flask, *α*-Fmoc-*ε*-Boc-l-lysin 4-methylcoumaryl-7-amide (1.818 g, 2.908 mmol), DMF (32 mL), and piperidine (8 mL) were added. The reaction mixture was stirred at ambient temperature for 30 min. The volatiles were removed under reduced pressure, and the residue was dissolved in ethyl acetate. The organic layer was washed with 10% Na_2_CO_3_ aq. and dried with Na_2_SO_4_. After filtration, the filtrate was evaporated to afford the crude product as a solid, which was washed with *t*-butyl methyl ether to afford amide **6** (0.879 g, 75% yield).

*ε*-Boc-l-lysin 4-methylcoumaryl-7-amide (**6**): ^1^H NMR (400 MHz, DMSO) δ = 7.83 (d, *J* = 2.0 Hz, 1 H, Ar-H), 7.72 (d, *J* = 8.0 Hz, 1 H, Ar-H), 7.56 (d, *J* = 2.0 Hz, 8.8 Hz, 1 H, Ar-H), 6.77 (s, 1 H, NH), 6.26 (d, *J* = 1.2 Hz, 1 H, Ar-H), 2.89 (s, 2 H, -C(O)NHC*H*_2_-), 2.40 (d, *J* = 1.2 Hz, 3 H, -CH_3_), 1.73–1.28 (m, 15 H, -CHC_3_*H*_6_-, -C(C*H*_3_)_3_) ppm. [8]

#### 3.2.5. Synthesis of *N*-Succinyl-l-phenylalanine *p*-nitroanilide

To a 50 mL round-bottomed flask, pyridine (15 mL), Et_3_N (0.1 mL, 0.7 mmol), anilide **4** (0.120 g, 0.7 mmol), and succinic anhydride (0.219 g, 2.19 mmol) were added. The reaction mixture was stirred at ambient temperature for 2 h. The volatiles were removed under reduced pressure, and the residue was dissolved in ethyl acetate. The organic layer was washed with 10% acetic acid aq. and then dried with Na_2_SO_4_. After filtration, the filtrate was evaporated to give the crude product as a solid, which was washed with diethyl ether to afford *N*-succinyl-l-phenylalanine *p*-nitroanilide (0.240 g, 89% yield). [23]

*N*-Succinyl-l-phenylalanine *p*-nitroanilide: ^1^H NMR (400 MHz, DMSO) δ = 10.66 (s, 1H, -N*H*C(O)-), 8.42 (d, *J* = 7.6 Hz, 1H, -N*H*CH(Bn)C(O)), 8.22 (d, *J* = 9.2 Hz, 2H, Ar-*H*), 7.84 (d, *J* = 9.2 Hz, 2H, Ar-*H*), 7.17–7.29 (m, 5H, -C_6_*H*_5_), 4.63–4.68 (m, 1H, -NHC*H*(Bn)C(O)), 3.05 (dd, *J* = 5.2 Hz, 13.6 Hz, 1H, Ph-C*H*_2_-), 2.88 (dd, *J* = 9.2 Hz, 13.6 Hz, 1H, Ph-C*H*_2_-), 2.326–2.383 (m, 4H, -C_2_*H*_4_CO_2_H) ppm.

#### 3.2.6. Synthesis of Boc-Protected Probe **Boc-P1**

To a 200 mL round-bottomed flask, *N*-succinyl-l-phenylalanine *p*-nitroanilide (0.212 g, 0.57 mmol), dichloromethane (35 mL), HOBt (0.0827 g, 0.615 mmol), and DIC (0.0827 g, 0.659 mmol) were added. After stirring for 10 min at ambient temperature, amide **6** (0.2298 g, 0.57 mmol) was added. The reaction mixture was stirred at ambient temperature for 70 min and thereby evaporated, and the obtained residue was dissolved in ethyl acetate. The organic layer was washed with *sat.* NaOH aq., 1 M HCl aq. and then with *sat*. NaHCO_3_ aq. and dried with Na_2_SO_4_. After filtration, the filtrate was dried under reduced pressure to afford the crude product as a solid, which was washed with *t*-butyl methyl ether to give Boc-protected P1 (0.351 g, 80% yield).

**Boc-P1**^1^H NMR (400 MHz, DMSO) δ = 10.63 (s, 1 H, N*H*), 10.41 (d, *J* = 17.2 Hz, 1 H, N*H*), 8.50 (s, 1 H, N*H*), 8.30 (s, 1 H, N*H*), 8.17 (dd, *J* = 24 Hz, 9.2 Hz, 2 H, Ar-*H*), 7.85 (t, *J* = 10.0 Hz, 2 H, Ar-*H*), 7.79 (d, *J* = 6.4 Hz, 1 H, Ar-*H*), 7.66 (d, *J* = 17.6 Hz,8.4 Hz, 1 H, Ar-*H*), 7.48 (t, *J* = 11 Hz, 1 H, Ar-*H*), 7.29–7.26 (m, 4 H, -C_6_*H*_5_), 7.27 (s, 1 H, -C_6_*H*_5_), 6.76 (s, 1 H, N*H*), 6.26 (d, *J* = 5.2 Hz, 1 H, Ar-*H*), 4.64 (s, 1 H, Ph-CH_2_C*H*-), 4.31 (s, 1 H, -C(O)NHC*H*-), 3.16–3.07 (m, 1 H, Ph-C*H*_2_-), 2.87 (s, 3 H, -NHC(O) C*H*_2_-, Ph-C*H*_2_-), 2.37 (d, *J* = 12 Hz, 7 H, -C*H*_3_, -C_2_*H*_4_C(O)-), 1.699–1.23 (m, 15 H, -C*H*_3_, -CHC_3_*H*_6_-, -C(C*H*_3_)_3_) ppm. HRMS (FAB, m/z) calc. 551.2294, found 551.2310.

#### 3.2.7. Synthesis of **P1**

To a 200 mL round-bottomed flask, **Boc-P1** (0.3784 g, 0.49 mmol) and dichloromethane (30 mL) were added. TFA (5 mL) was added to the solution, and the reaction mixture was stirred at ambient temperature for 3 h. After removal of volatiles, the obtained crude product was washed with *t*-butyl methyl ether and dried in vacuo to afford **P1** as a TFA adduct (0.105 g, 32% yield).

**P1**^1^H NMR (400 MHz, DMSO) δ = 10.57 (d, *J* = 8.0 Hz, 1 H, N*H*), 10.37 (d, *J* = 22 Hz, 1 H, N*H*), 8.46 (d, *J* = 8.4 Hz, 1 H, N*H*), 8.29 (d, *J* = 7.2 Hz, 1 H, N*H*), 8.37–8.164 (m, 2 H, Ar-*H*), 7.87–7.79 (m, 3 H, Ar-*H*), 7.67 (t, *J* = 9.8 Hz, 1 H, Ar-*H*), 7.61 (s, 2 H, N*H*_2_), 7.47 (t, *J* = 8.0 Hz, 1 H, Ar-*H*), 7.29–7.26 (m, 4 H, -C_6_*H*_5_), 7.19 (s, 1 H, -C_6_*H*_5_), 6.26 (d, *J* = 1.2 Hz, 1 H, Ar-*H*), 4.64 (s, 1 H, Ph-CH_2_C*H*-), 4.35 (s, 1 H, -NHC(O)C*H*-), 3.10 (dd, *J* = 14 Hz, 5.2 Hz, 1 H, Ph-C*H*_2_-), 2.87 (t, *J* = 12.6 Hz, 1 H, Ar-*H*), 2.76 (s, 2 H, -C(O)NHC*H*_2_-), 2.37 (d, *J* = 16 Hz, 7 H, -C*H*_3_, -C_2_*H*_4_C(O)-), 1.74–1.32 (m, 6 H, -CHC_3_*H*_6_-) ppm. ^13^C NMR δ (CDCl_3_) = 18.4, 22.9, 27.0, 27.3, 30.9, 31.4, 37.6, 38.9, 49.2, 54.0, 55.7, 106.25, 112.7, 115.5, 115.8, 116.3, 119.5, 125.3, 126.2, 126.9, 128.6, 129.6, 138.0, 142.7, 145.4, 153.5, 154.0, 158.2, 158.6, 160.5, 171.8, 172.0, 172.4, 172.5 ppm. HRMS (FAB, m/z) calc. 551.2294, found 551.2310.

#### 3.2.8. Synthesis of l-lysin 4-methylcoumaryl-7-amide (**1d**)

To a 200 mL round-bottomed flask, *ε*-Boc-l-lysine 4-methylcoumaryl-7-amide (**6**) (0.137 g, 0.34 mmol) and dichloromethane (30 mL) were added. TFA (5 mL) was added to the solution, and the reaction mixture was stirred at ambient temperature for 3 h. After removal of volatiles, the obtained crude product was washed with *t*-butyl methyl ether and dried in vacuo to afford Lys-AMC **1d** (0.072 g, 70% yield)

Lys-AMC **1d** ^1^H NMR (400 MHz, DMSO) δ = 8.4 (brs, 2 H, N*H*_2_), 7.85–7.77 (m, 4 H, N*H*_2_, Ar-*H*), 7.58 (dd, *J* = 8.8 Hz, 2.0 Hz, 1 H, Ar-*H*), 6.32 (d, *J* = 1.2 Hz, 1 H, Ar-*H*), 5.58 (d, *J* = 8.0 Hz, 1 H, Ar-*H*), 4.09–4.06 (brm, 1 H, -NHC(O)C*H*-), 2.81–2.74 (brm, 2 H, -C*H*_2_NH_2_), 2.41 (d, *J* = 1.2 Hz, 3 H, -C*H*_3_), 1.90–1.80 (m, 1 H, -CHC_3_*H*_6_-), 1.73–1.68 (m, 1 H, -CHC_3_*H*_6_-), 1.62–1.40 (m, 2 H, -CHC_3_*H*_6_-), 1.30–1.02 (m, 2 H, -CHC_3_*H*_6_-) ppm.

#### 3.2.9. Synthesis of AMC-lys(Boc)-suc

To a 50 mL round-bottomed flask, pyridine (15 mL), Et_3_N (0.1 mL, 0.7 mmol), *ε*-Boc-l-lysine 4-methylcoumaryl-7-amide (**6**) (0.15 g, 0.37 mmol), and succinic anhydride (0.219 g, 2.19 mmol) were added. The reaction mixture was stirred at ambient temperature for 2 h. The volatiles were removed under reduced pressure, and the residue was dissolved in ethyl acetate. The organic layer was washed with 10% acetic acid aq. and then dried with Na_2_SO_4_. After filtration, the filtrate was evaporated to give the crude product as a solid, which was washed with diethyl ether to afford AMC-lys(Boc)-suc (0.024 g, 13% yield).

AMC-lys(Boc)-suc ^1^H NMR (400 MHz, DMSO) δ = 10.4 (s, 1 H, N*H*), 8.23 (d, *J* = 7.2 Hz, 1 H, N*H*), 7.81 (s, 1 H, Ar-*H*), 7.71 (d, *J* = 8.8 Hz, 1 H, Ar-*H*), 7.55 (d, *J* = 9.6 Hz, 1 H, Ar-*H*), 6.77–6.74 (m, 1 H, N*H*), 6.26 (d, *J* = 1.2 Hz, 1 H, Ar-*H*),5.56 (d, *J* = 8.4 Hz, 1 H, N*H*), 4.37–4.32 (brm, 1 H, -NHC(O)C*H*-), 2.91–2.86 (m, 2 H, -C(O)NHC*H*_2_-), 2.40 (s, 7 H, -C*H*_3_, -C_2_*H*_4_C(O)-), 1.74–1.68 (m, 2 H, -CHC_3_*H*_6_-), 1.63–1.57 (m, 2 H, -CHC_3_*H*_6_-), 1.37–1.20 (m, 10 H, -C*H*_3_, -CHC_3_*H*_6_-, -C(C*H*_3_)_3_) ppm.

#### 3.2.10. Synthesis of AMC-lys-suc (Fragment Probe **1e**)

To a 50 mL round-bottomed flask, AMC-lys(Boc)-suc (0.044 g, 0.09 mmol) and dichloromethane (10 mL) were added. TFA (2 mL) was added to the solution, and the reaction mixture was stirred at ambient temperature for 3 h. After removal of volatiles, the obtained crude product was washed with *t*-butyl methyl ether and dried in vacuo to afford AMC-lys-suc **1e** (0.036 g, 85% yield)

AMC-lys-suc **1e** ^1^H NMR (400 MHz, DMSO) δ = 10.4 (s, 1 H, N*H*), 8.24 (d, *J* = 8.0 Hz, 1 H, N*H*), 7.79 (d, *J* = 2.0 Hz, 1 H, N*H*), 7.73 (d, *J* = 8.4 Hz, 2 H, Ar-*H*), 7.51 (d, *J* = 8.8 Hz,2.4 Hz, 1 H, Ar-*H*), 6.28 (s, 1 H, Ar-*H*), 5.57 (d, *J* = 8.0 Hz, 1 H, N*H*), 4.43–4.37 (brm, 1 H, -NHC(O)C*H*-), 2.77 (t, *J* = 7.8 Hz, 2 H, -C*H*_2_NH_2_), 2.43–2.38 (m, 7 H, -C*H*_3_, -C_2_*H*_4_C(O)-), 1.74–1.68 (m, 2 H, -CHC_3_*H*_6_-), 1.64–1.50 (m, 3 H, -CHC_3_*H*_6_-) 1.37–1.05 (m, 1 H, -CHC_3_*H*_6_-) ppm.

### 3.3. Preparation of Solutions

#### 3.3.1. pH 8.0 Tris-HCl Buffer Solution

2-Amino-2-hydroxymethyl-1,3-propanediol (1.938 g) was dissolved in 100 mL of distilled water. The pH of the solution was adjusted to 8.0 by the careful addition of 1 M HCl aq. The final volume of the solution was brought to 200 mL with distilled water.

#### 3.3.2. pH 7.4 PBS Buffer Solution

Potassium dihydrogen phosphate (0.06 g, 0.6 mmol), potassium chloride (0.06 g, 0.81 mmol), disodium hydrogen phosphate 12-water (0.87 g, 2.4 mmol), and sodium chloride (2.4 g, 41.1 mmol) were dissolved in distilled water. After confirming the pH of the solution was 7.4, the final volume of the solution was brought to 300 mL with distilled water.

#### 3.3.3. pH 2.2 Glycine-HCl Buffer Solution

Glycine (1.5 g, 20 mmol) was dissolved in 100 mL of distilled water. Sodium chloride (1.18 g, 20 mmol) was then added to the solution; the pH of the solution was adjusted to 2.2 with 1 M HCl aq. The final volume of the solution was brought to 200 mL with distilled water.

#### 3.3.4. 0.59 mM **P1** Solution

**P1** (0.0046 g, 5.9 µmol) was dissolved in DMSO (0.8 mL) and methanol (2 mL). The final volume of the solution was brought to 10 mL with distilled water.

#### 3.3.5. 0.59 mM *p*-Nitroaniline and AMC Solution

*p*-Nitroaniline (0.0008 g, 5.9 μmol) or AMC (0.001 g, 5.9 μmol) was dissolved in DMSO (0.8 mL) and methanol (2 mL). The final volume of the solution was brought to 10 mL with distilled water.

#### 3.3.6. 0.59 mM Solution of Fragment Probe **1b**–**e**

Fragment probe **1b** (0.0018 g, 5.9 μmol) or **1c** (0.0017 g, 5.9 μmol) was dissolved in DMSO (0.8 mL) and methanol (2 mL). The final volume of the solution was brought to 10 mL with distilled water. **1d** (0.0017 g, 4.4 μmol) was dissolved in DMSO (0.6 mL) and methanol (1.5 mL). The final volume of the solution was brought to 10 mL with distilled water. **1e** (0.0012 g, 42.95 μmol) was dissolved in DMSO (0.4 mL) and methanol (1 mL). The final volume of the solution was brought to 5 mL with distilled water.

#### 3.3.7. 36 µM **P1** in PBS Buffer Solution

**P1** (0.0016 g, 2.0 µmol) was dissolved in DMF (0.8 mL), and then the volume of the solution was brought to 20 mL with pH 7.4 PBS buffer solution. The obtained solution (3.6 mL) was diluted to a pH 7.4 PBS buffer solution, and the final volume of the solution was brought to 10 mL with the buffer solution.

#### 3.3.8. 36 µM Solution of Fragment Probe **1b** and **1e** in PBS

Fragment probe **1b** (0.0040 g, 0.01 mmol) was dissolved in DMF (0.4 mL), and the volume of the solution was brought to 20 mL with pH 7.4 PBS buffer solution. The obtained solution (0.36 mL) was diluted to a pH 7.4 PBS buffer solution, and the final volume of the solution was brought to 10 mL with pH 7.4 PBS buffer solution. **1e** (0.0030 g, 0.01 mmol) was independently dissolved in DMF (0.2 mL), and the volume of the solution was brought to 5.0 mL with pH 7.4 PBS buffer solution. The obtained solution (0.36 mL) was diluted to a pH 7.4 PBS buffer solution, and the final volume of the solution was brought to 10 mL with the buffer solution.

#### 3.3.9. Solution of α-Chymotrypsin, Pepsin, Elastase, Proteinase K, and Papain in HCl

α-chymotrypsin (0.01 g), pepsin (0.0012 g), elastase (0.0023 g), proteinase K (0.0062 g) or papain (0.1 g) was dissolved in 1.0 mM HCl aq. (1 mL). Nattokinase (0.208 g) was dissolved in 1.0 mM HCl aq. (0.25 mL). These solutions were stored in an ice bath before use.

#### 3.3.10. Solution of Trypsin in PBS Buffer

Trypsin (0.01 g) was dissolved in pH 7.4 PBS buffer solution (10 mL). The obtained solution (0.075 mL) was diluted to a pH 7.4 PBS buffer solution, and the final volume of the solution was brought to 10 mL with the buffer solution.

### 3.4. Reaction of Molecular Probes with Proteases

The reaction of **P1** with proteases was monitored by using both a test solution (with protease) and a blank solution (without protease) for control experiments. The reactions were performed at ambient temperature.

#### 3.4.1. Reaction of **P1** with α-Chymotrypsin

The 0.59 mM **P1** solution (0.13 mL) was diluted to pH 8.0 Tris-HCl buffer (3 mL). To the solution was added CaCl_2_ aq. (0.04 mL), the α-chymotrypsin solution above (0.05 mL) for the test sample or 1 mM HCl aq. (0.05 mL) for the blank sample. After 5 min, UV-vis and fluorescent emission spectra of both the test sample and the blank sample were recorded.

#### 3.4.2. Reaction of **P1** with Trypsin

The 36 µM **P1** solution (2.0 mL) was mixed with pH 7.4 PBS buffer solution (1 mL) to obtain the blank sample. To the 36 µM **Probe 2** solution (2 mL), trypsin solution (above 1 mL) was added to obtain the test sample. After 5 min, UV-vis and fluorescent emission spectra of both the test sample and the blank sample were recorded.

#### 3.4.3. Reaction of **P1** with Proteases

##### Preparation of Test Solutions

Test solutions with α-chymotrypsin, proteinase K, elastase, papain, and nattokinase: The **P1** solution (0.13 mL) was mixed with pH 8.0 Tris-HCl buffer solution (3 mL) and CaCl_2_ aq. (0.04 mL). The protease solution (0.05 mL) was added to the solution to obtain the test sample.

Test solutions with trypsin: The **P1** solution (2.0 mL) was mixed with the trypsin solution (1 mL) to obtain the test sample.

Test solutions with pepsin: The **P1** solution (0.13 mL) was mixed with a pH 2.2 glycine-HCl buffer solution (3 mL) and CaCl_2_ aq. (0.04 mL). The pepsin solution (0.05 mL) was added to the obtained solution to obtain the test sample.

##### Determination of *p*-Nitroaniline Concentration after 5 min

Five minutes after the test sample was prepared, some of it was injected into the HPLC machine. The concentration of *p*-nitroaniline was calculated from the obtained peak area (R*_t_* = 3.8 min) and the calibration curves (Supplementary Materials).

##### Time Course of *p*-Nitroaniline Concentration

After the test sample was prepared, some of it was injected into the HPLC spectrometer after 0.5, 1, 3, 5, 7, 9, and 11 min. Concentrations of *p*-nitroaniline at each time were calculated from the obtained peak area (Rt = 3.8 min) and the calibration curves.

##### Determination of AMC Concentration

Five minutes after the test sample was prepared, its fluorescence emission spectrum was recorded. The concentration of AMC was calculated from the obtained fluorescent intensity and the calibration curves.

##### Time Course of AMC Concentration

After the test sample was prepared, its fluorescent intensity (ex: 360 nm; em: 441 nm) was measured every 0.8 s. The obtained fluorescent intensity was plotted to obtain time-course curves.

#### 3.4.4. Reaction of **P1** Fragment Probes

The enzymatic reactions were performed under the conditions described above by using the **1b**–**e** solutions instead of the **P1** solution.

## 4. Conclusions

A new chromogenic and fluorescent molecular probe (**P1**) for detecting proteases was designed and synthesized. **P1** reported the presence of α-chymotrypsin, trypsin, papain, and nattokinase by releasing *p*-nitroaniline and/or AMC. The type of protease can be distinguished by the differences in the balances of the obtained *p*-nitroaniline and AMC. In addition, the time courses of the reactions could report the difference in substrate selectivity between α-chymotrypsin and nattokinase. Further studies now underway include synthesizing other probes bearing various amino acid moieties and categorizing proteases by using these molecular probes.

## Data Availability

The data presented in this study are available on request from the corresponding author.

## Supplementary Materials

The following are available online, Figure S1–S4: calibration curves of *p*-nitroaniline and AMC, Figure S5–S14: NMR and MS spectra of substrates, Figure S15: UV-vis spectra of proteases.

## Sample Availability

Samples of the compounds are not available from the authors.
