# Supplementary material for: A Molecular Probe with Both Chromogenic and Fluorescent Units for Detecting Serine Proteases
Source: Molecules. 2021 Jan 18;26(2):482. doi: 10.3390/molecules26020482 (PMC7831087; doi:10.3390/molecules26020482)
Supplement: Supplementary file 1 [file molecules-26-00482-s001.pdf]

Supporting Information of

A Molecular Probe with Both Chromogenic and Fluorescent Units for Detecting Serine Proteases

Kirara Ishida, Yushi Nakamura, Tetsuo Ohta, and Yohei Oe*

Department of Biomedical Sciences and Informatics, Faculty of Life and Medical Sciences, Doshisha University; yoe@mail.doshisha.ac.jp

1. **Calibration Curves**
2. **NMR Spectra**
3. **UV-vis spectra of Proteases**

1. Calibration curves

1.1. Preparation of calibration curve of AMC in Tris buffer solutions

AMC (0.012 g, 0.08 mmol) was dissolved in DMSO (1.6 mL) and methanol (4 mL), and the volume of the solution was brought to 20 mL with distilled water. Obtained 4 mM AMC solution was then diluted to 0.024, 0.124, and 0.223 mM with distilled water. The prepared AMC solution (0.13 mL) was mixed with pH 8.0 Tris-HCl buffer solution (3 mL), $CaCl_2$ *aq.* (0.04 mL), 1 mM HCl *aq.* (0.05 mL), and the fluorescent-emission spectra of each solution was recorded. Fluorescent intensity was plotted against concentration to give a calibration curve, which was found to be linear in according with the equation $y = 3.00\times 10^{-5}x - 0.00001$ with R^2=1.0000.

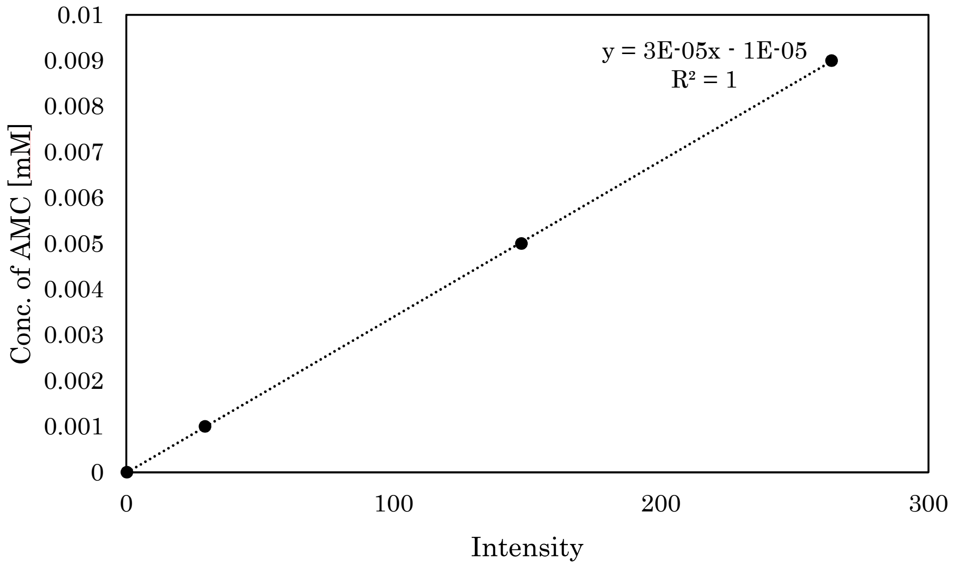

Figure S1 Calibration curve of AMC in Tris buffer solutions

1.2. Preparation of calibration curve of AMC in PBS buffer solutions

AMC (0.012 g, 0.08 mmol) was dissolved in DMF (4.0 mL), and the volume of the solution was brought to 20 mL with pH 7.4 PBS buffer solution. The obtained 5.0 mM AMC solution was then diluted to 1.5, , 7.5, and 13.5 μM. The prepared AMC solution (2.0 mL) was then mixed with pH 7.4 PBS buffer solution (1.0 mL), and the fluorescent-emission spectra of each solution was recorded. Plotting fluorescent intensity versus concentration gave a calibration curve, which was found to be linear in according with the equation $y = 3.00\times 10^{-5}x - 0.00008$ with R^2=0.9996.

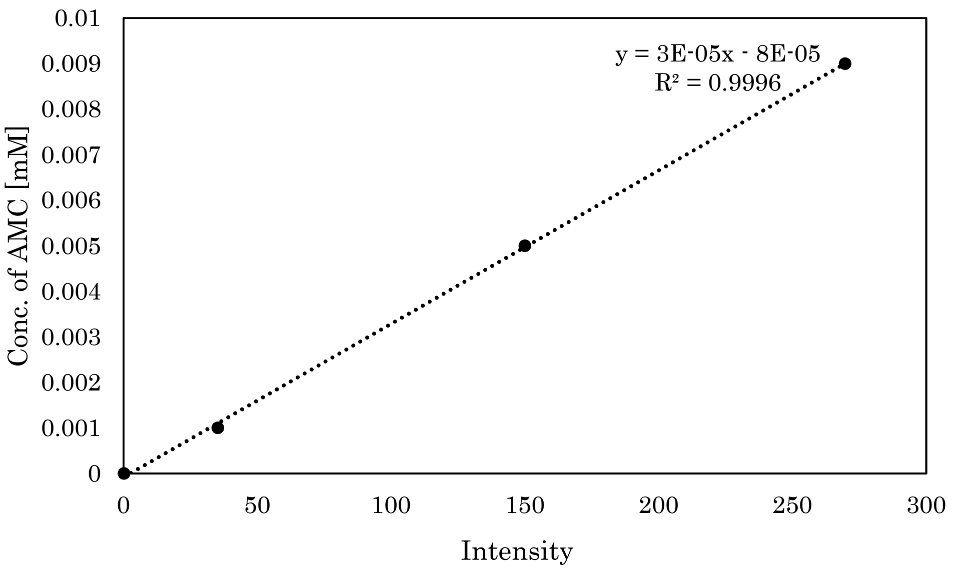

Figure S2 Calibration curve of AMC in PBS buffer solutions

1.3. Preparation of calibration curve of *p*-nitroaniline for the reaction with chymotrypsin

p-nitroaniline (0.011 g, 0.08 mmol) was dissolved in methanol (4 mL) and DMSO (1.6 mL), and the volume of the solution was brought to 20 mL with distilled water. The obtained 4-mM *p*-nitroaniline solution was diluted to 0.1, 0.2, 0.4, and 0.6 mM with distilled water. These solutions were independently mixed with pH 8.0 Tris-HCl buffer solution (3 mL), $CaCl_2$ *aq.* (0.04 mL), and 1-mM HCl *aq.* (0.05 mL), and they were then injected into the HPLC machine. Plotting the obtained peak areas of *p*-nitroaniline (R_t = 3.8 min) versus concentrations gave the calibration curve, which was found to be linear in according with the equation $y = 1.00\times 10^6x - 187.98$ with $R^2 = 0.9948$.

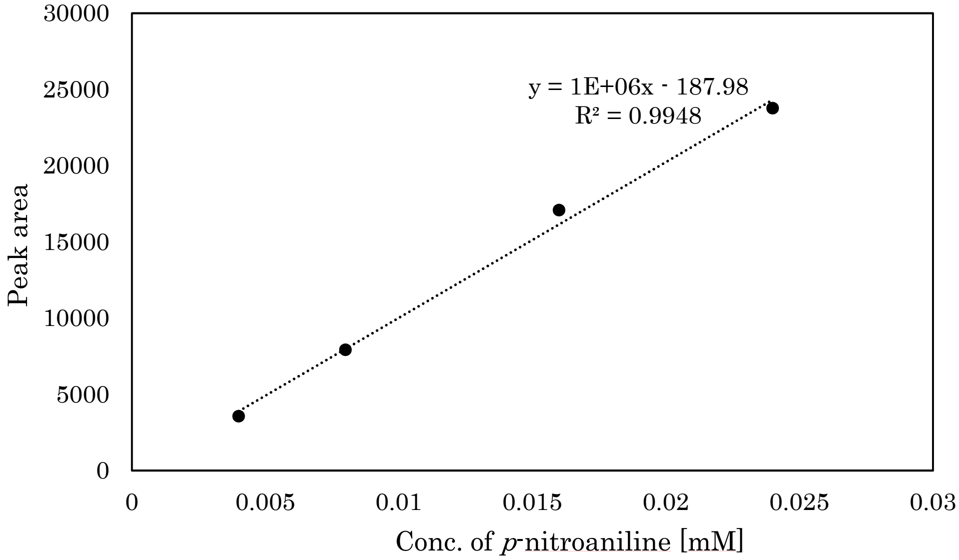

Figure S3 Calibration curve of *p*-nitroaniline for the reaction with chymotrypsin

1.4. Preparation of calibration curve of *p*-nitroaniline for the reaction with trypsin

p-nitroaniline (0.014 g, 0.1 mmol) was dissolved in DMF (4.0 mL), and volume of the solution was brought to 20 mL with pH 7.4 PBS buffer solution. The obtained 5.0-mM *p*-nitroaniline solution was then diluted to 0, 20 40, 60, and 80 mM with the buffer solution. The prepared *p*-nitroaniline solutions (2.0 mL) were independently mixed with pH 7.4 PBS buffer solution (1.0 mL), which was injected into the HPLC machine. Plotting the obtained peak areas of *p*-nitroaniline (R_t = 3.8 min) versus concentrations gave the calibration curve, which was found to be linear in according with the equation $y = 1.00\times 10^6 x + 849$ with $R^2 = 0.9986$.

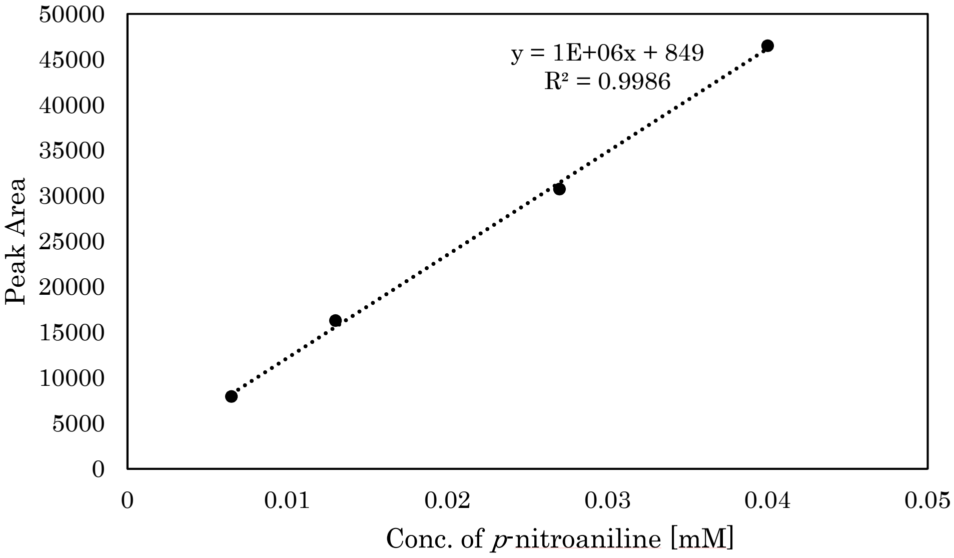

Figure 4 Calibration curve of *p*-nitroaniline for the reaction with trypsin

2. NMR and MS spectra

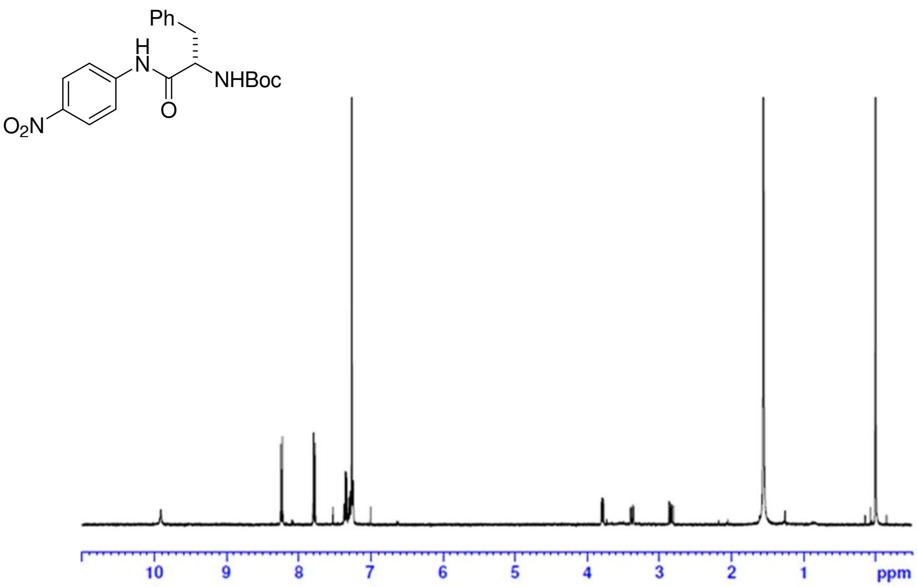

Figure S5 ^{1}H NMR spectra of *N*-(Boc)-L-phenylalanine *p*-nitroanilide (**3**).

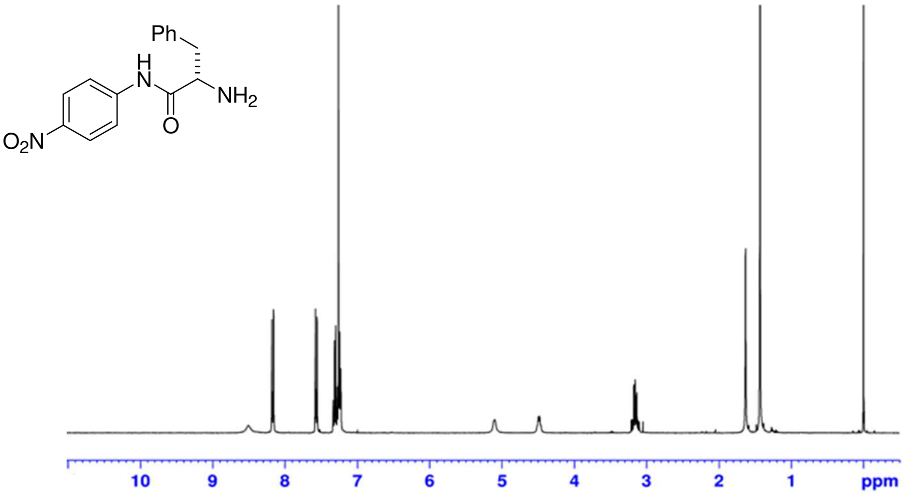

Figure S6 ^{1}H NMR spectra of L-phenylalanine *p*-nitroanilide (**4**).

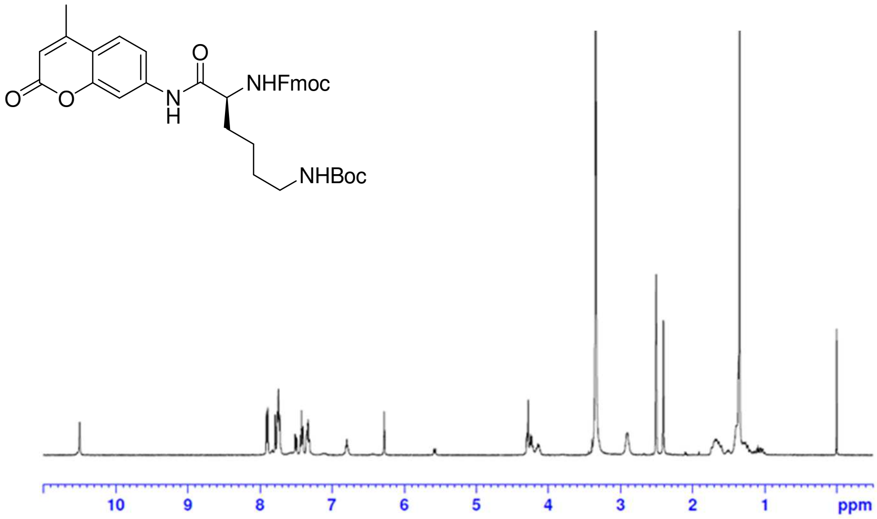

Figure S7 ^{1}H NMR spectra of *ε*-Boc-L-lysin 4-methylcoumaryl-7-amide (**6**).

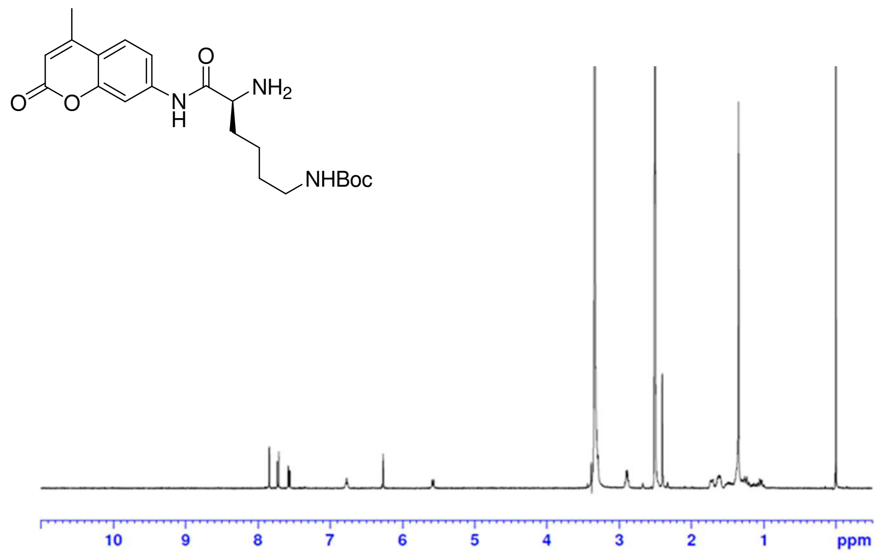

Figure S8 ^{1}H NMR spectra of *α*-Fmoc-*ε*-Boc-L-lysin 4-methylcoumaryl-7-amide.

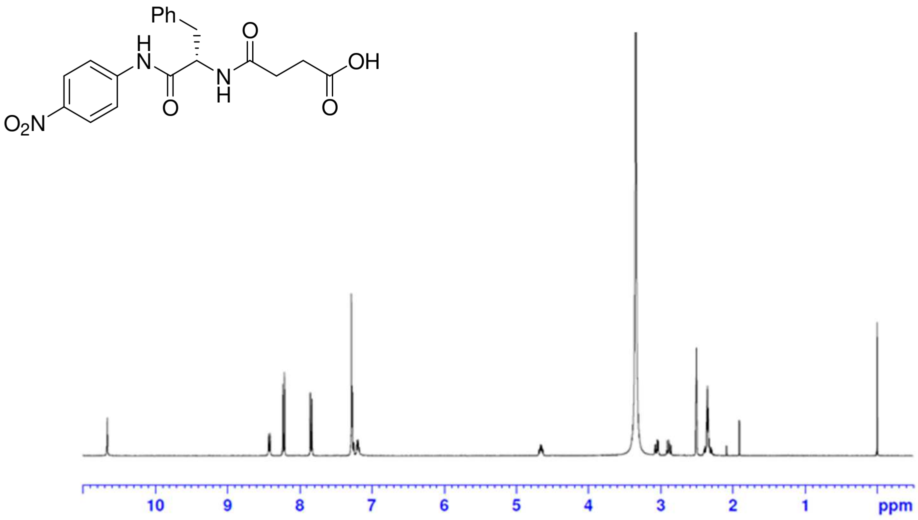

Figure S9 ^{1}H NMR spectra of *N*-succinyl-L-phenylalanine *p*-nitroanilide.

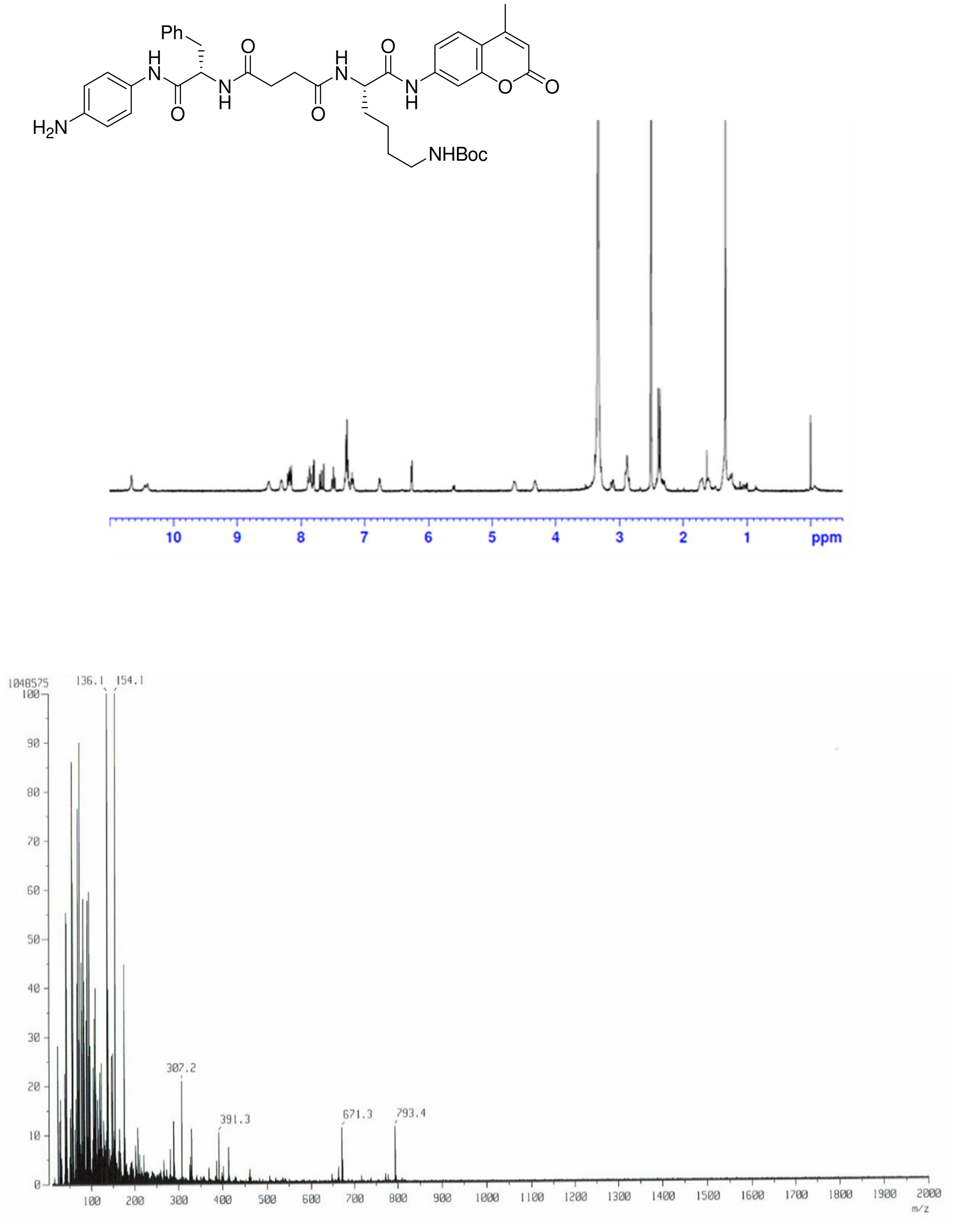

Figure S10 ^{1}H NMR and FAB-MS spectra of **Boc-P1**.

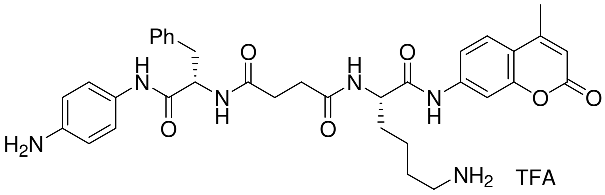
TFA

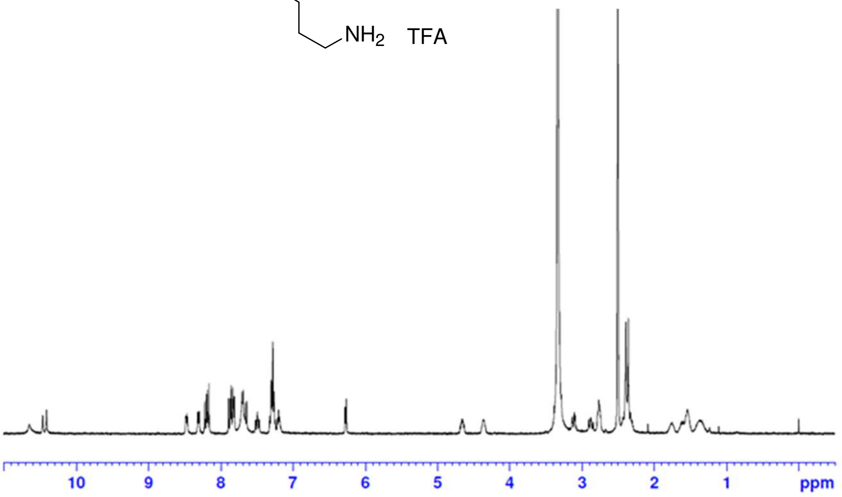
10
9
8
7
6
5
4
3
2
1
ppm

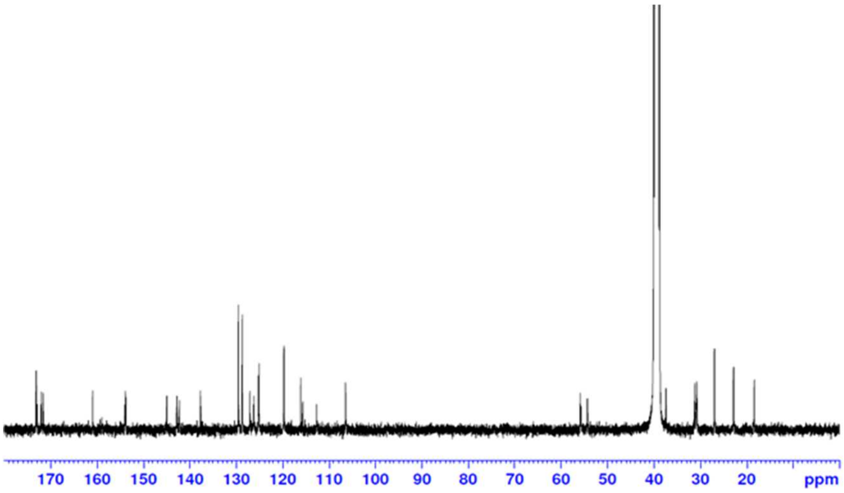
170
160
150
140
130
120
110
100
90
80
70
60
50
40
30
20
ppm

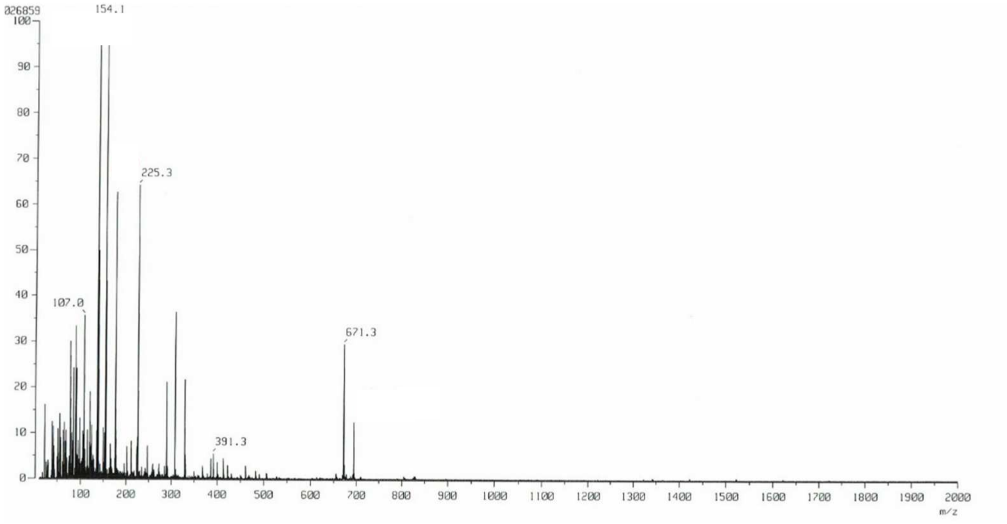

Figure S11 ^{1}H NMR and ^{13}C NMR, FAB-MS spectra of **P1**.

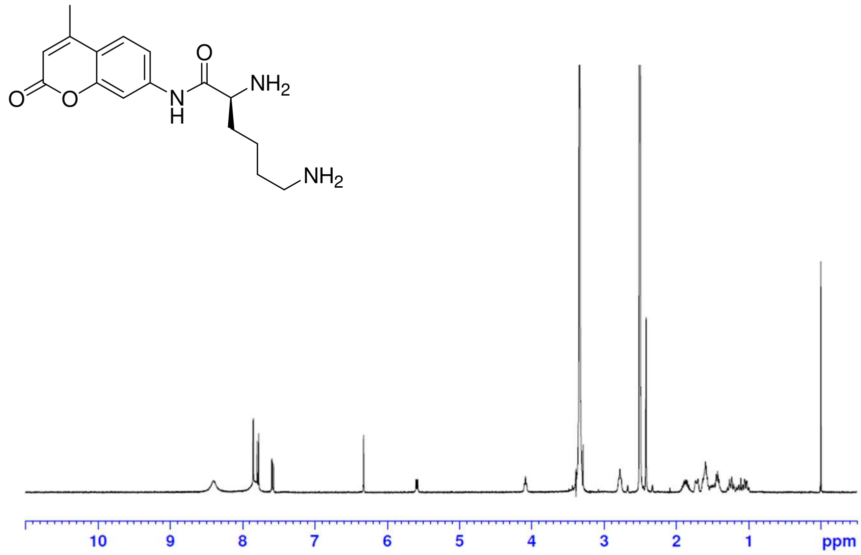

Figure S12 ^{1}H NMR spectra of lys-AMC **7**.

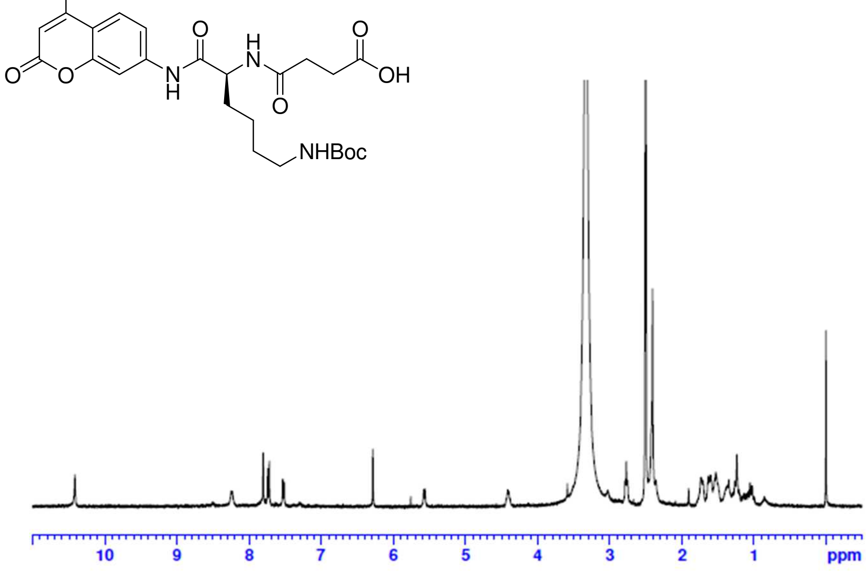

Figure S13 ^{1}H NMR spectra of AMC-lys(Boc)-suc .

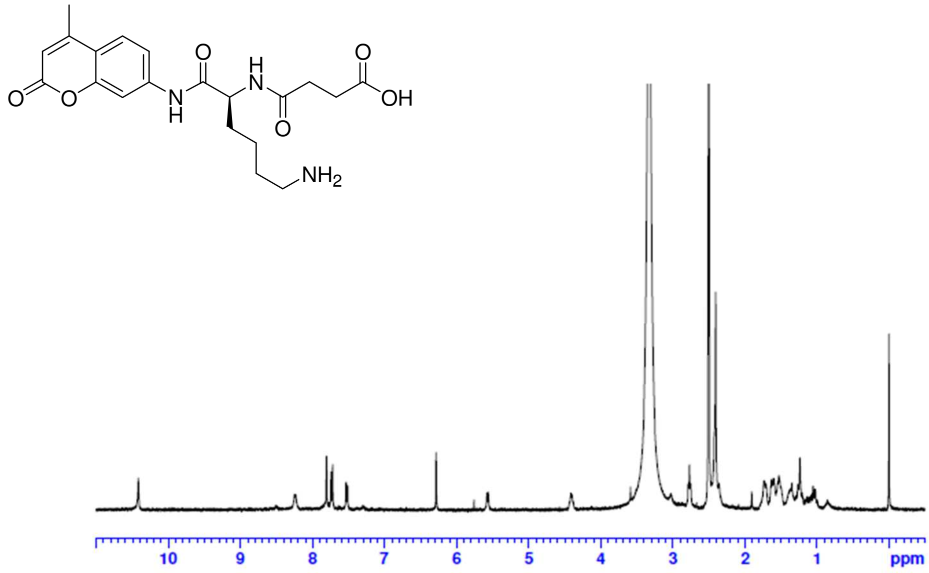

Figure S14 ^{1}H NMR spectra of AMC-lys-suc **8**.

3. UV-vis spectra of proteases.

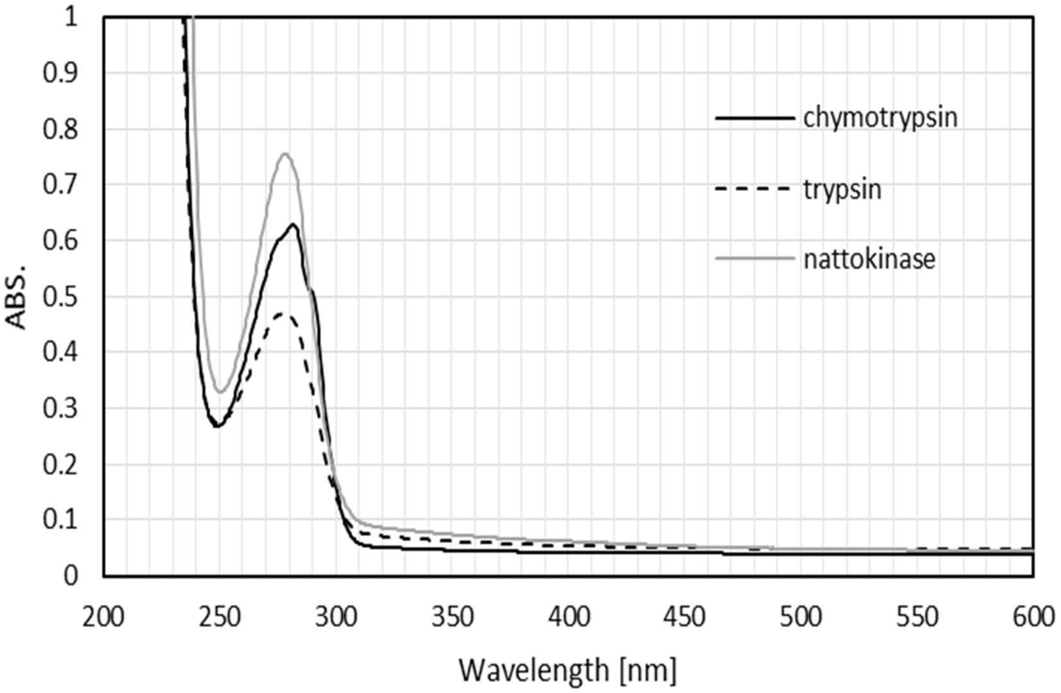

Figure S15 UV-vis spectra of proteases.
